# Twice weekly hemodialysis is safe at the beginning of kidney replacement therapy: the experience of the Nephrology Department at Hedi Chaker University Hospital, Sfax, south of Tunisia

**DOI:** 10.11604/pamj.2020.35.129.20285

**Published:** 2020-04-20

**Authors:** Hanen Chaker, Faiçal Jarraya, Salma Toumi, Khawla Kammoun, Yosra Mejdoub, Hichem Mahfoudh, Soumaya Yaich, Mohamed Ben Hmida

**Affiliations:** 1 Nephrology Department, Hedi Chaker University Hospital and UR 12ES14 Faculty of Medicine, Sfax, Tunisia; 2 Faculty of Medicine, Community Medicine Department, Hedi Chaker University Hospital, Sfax, Tunisia

**Keywords:** Renal replacement therapy, hemodialysis, mortality

## Abstract

We re-examine the infrequent paradigm of a biweekly dialysis at the start of renal replacement therapy. The current method is to launch hemodialysis among patients using a ‘full-dose’ posology three times a week. As a matter of fact, recent data has suggested that frequent hemodialysis leads to high mortality at the onset of dialysis. The aim of our study is to show the factors affecting early mortality especially the hemodialysis frequency. We undertook an observational study in the hemodialysis unit of Sfax University Hospital (south Tunisia). We enrolled the incident patients during one year. Baseline demographic and clinical characteristics of patients were noted. The survival status of each patient is observed at 6 months after the onset of hemodialysis. We analyzed the factors associated with mortality, especially the hemodialysis frequency (twice or thrice weekly hemodialysis regimen). We enrolled 88 patients with mean age of 56 ± 18 years old. Thirty patients underwent twice weekly dialysis (Group 1) and 58 patients underwent thrice weekly dialysis (Group 2). The mortality at 6 months was similar in the 2 groups (the rate of death = 30% in group 1 vs 13.8% in group 2, p = 0.07). However, the mortality was lower in the group with preserved residual diuresis (35.3% vs 64.7% in the group without residual diuresis, p = 0.02). The mortality was higher in diabetes patients (64.7% vs 35.5%, p = 0.02). It was concluded that twice or threefold weekly treatment have some considerable similar outcomes on the patients survival (at 6 months).

## Introduction

High mortality in incident patients who start hemodialysis (HD) has been reported in several publications [1, 2]. Strangely enough, this mortality is high in low, middle and high-income countries. Indeed, at the international level, the early hemodialysis period is life-threatening for patients in many countries but with substantial differences in mortality between them. Hence, the efforts to improve end results should focus on both the transition period and the first few months of dialysis [2]. Considering the high mortality rates during the first 6 months of hemodialysis and the survival benefits of preserved native kidney function, an initiation of the treatment twice a week ("infrequent hemodialysis") complemented with an incremental increase in frequency over time may promote an opportunity to optimize patient survival [3]. So far, this infrequent paradigm has been controversial for authors who are for and against “twice weekly dialysis. After the IDEAL study [4], although the problem of the early against the late onset of renal replacement therapy has already been resolved between supporters and opponents, it is still uncertain whether twice or thrice weekly hemodialysis has better results at the outset. It is in this context that we have tried to review the literature and capitalize on our experience in this domain so as to draw conclusions that could help nephrologists in their medical care.

## Methods

We undertook an observational study in the hemodialysis unit of Sfax University Hospital (South Tunisia). We enrolled the incident patients during one year. Baseline demographic and clinical characteristics including age, diabetes, hypertension, residual diuresis, and biological data namely creatinine, hemoglobin average were collected.

At the start of renal replacement therapy and for economic constraints, the patients with no social security starts dialysis in the hospital once weekly. They are switched to 2 sessions only if they develop a state of overload or hyperkalemia requiring further sessions in emergency. By contrast, the patients who had social security were provided with dialysis in the private sector three times a week at the onset of hemodialysis. The evolution was surveyed for patients who had dialysis twice weekly dialysis in the hospital and patients who had thrice weekly dialysis in the private sector. Mortality at 6 months was observed.

The data was collected and analyzed using the 20^th^ version Statistical Package for Social Sciences (SPSS) computer software. The categorical variables were expressed as a percentage. For the continuous variables, we verified the normality of the distribution using the Kolmogorov-Smirnov test and the Shapiro-Wilk test. Eventually, an estimate of the means with their standard deviations and the median with min and max was performed. The comparison between the two categorical variables was achieved by Pearson's "chi2" test when the conditions were fulfilled; otherwise Fisher's exact test was used. The student's test was used to compare the two means when the distribution is Gaussian while the non-parametric test of Mann-Whitney U was applied when the distribution is not Gaussian.

## Results

We enrolled 88 patients with mean age of 56 ± 8 years old. Thirty patients underwent twice weekly dialysis (Group 1) and 58 patients underwent thrice weekly dialysis (Group 2). The 2 groups were comparable for age (53.9 ± 20 years old vs 58.1 ± 16.4, p = 0.2) and for diabetes as well (43.3% vs 39.7%, p = 0.7). They were also comparable for hypertension (56.7% vs 70.7%, p = 0.2), preserved residual diuresis (70% vs 53.4%, p = 0.1), hemoglobin average (7.7 g/dl vs 7.9 g/dl, p = 0.6) and creatinine clearance (6.9 ml/mn vs 6.4 ml/mn, p = 0.1) (Table 1). The mortality at 6 months was similar in the 2 groups (the rate of death = 30% in group 1 vs 13.8% in group 2, p = 0.07). However the mortality was lower in the group with preserved residual diuresis (35.3% vs 64.7% in the group without residual diuresis, p = 0.02). The mortality was higher in diabetes patients (64.7% vs 35.5%, p = 0.02) (Table 2).

**Table 1:** Baseline demographic and clinical characteristics in HD patients based on HD frequency

	Twice weekly HD N= 30	Thrice weekly HD N=58	P value
Age	53.9±20	58.19±16.4	0.2
Diabetes	13 (43.3%)	23 (39.7%)	0.7
Hypertension	17 (56.7%)	41 (70.7%)	0.2
Preserved Residual diuresis	21 (70%)	31 (53.4%)	0.1
Hemoglobin (g/dl)	7.7±2	7.9±1.9	0.6
Creatinine clearance (ml/mn)	6.9±1.7	6.4±1.5	0.1

**Table 2:** Incidence of deaths based on status of HD frequency, preserved residual diuresis and diabetes

		Mortality N of events (%)	P value
HD frequency	Twice weekly	9 (30%)	0.07
	Thrice weekly	8 (13.8%)	
Preserved Residual diuresis	Yes	6 (35.3%)	0.02
	No	11 (64.7%)	
Diabetes	Yes	11 (64.7%)	0.02
	No	6 (353%)	

## Discussion

In this part of our study, we will prove that twice and thrice weekly regimens have nearly similar outcomes at 6 months.

**Why is the mortality risk high at the beginning of the dialysis?** Actually, in 2007, the mortality risk was the highest in the first 120 days after hemodialysis initiation in DOPPS. Indeed, inadequate pre-dialysis nephrology treatment was strongly associated with mortality during this period. In particular, older age, catheter vascular access, albumin <3.5g/dl, phosphorus <3.5mg/dl, cancer and congestive heart failure all were involved in the death rate increase [5]. In our cohort, diabetes was associated with high mortality at 6 months (p = 0.02). Amazingly, residual kidney function (RKF) wasn´t studied as an influential factor on mortality. Given the importance of the residual renal function (RRF) preservation in conservative therapy, it is inconsistent to disregard the contribution of RRF when patients start hemodialysis, especially when it is frequently regarded as a peritoneal dialysis [6]. In effect, the full loss of residual renal function causes higher mortality levels in dialysis patients [7]. In our cohort, preserved residual diuresis was associated with lower mortality at 6 months (p = 0.02).

**Preserving renal residual function is crucial:** in actual fact, there is a growing focus on preserving RRF. Observational studies have shown that the preservation of RRF in dialysis patients is a prognostic and an independent factor in the survival and the quality of life of the patient [8, 9]. Conversely, augmenting the dialysis dose failed to have an impact on the mortality of dialysis patients [10]. Indeed, the recurrent hemodialysis might alter volume status, blood pressure, and the concentration of osmotically active solutes, each of which might affect RKF [11]. Hence, during the transition from Stage 5 of the chronic kidney disease, to the end-stage renal disease (ESRD) requiring dialysis, the question is to initiate hemodialysis on a “full-dose” of thrice-weekly regimen even among patients with substantial residual renal function. However, emerging data suggest that frequent hemodialysis accelerates residual renal function decline and infrequent regimens may provide better preservation of native kidney function [3].

**Which schedule is suitable at the initiation of hemodialysis? I**n our cohort, the mortality at 6 months was similar in the group 1 of twice weekly regimen compared with the group 2 of thrice weekly (the rate of death = 30% in group 1 vs 13.8% in group 2, p = 0.07). We specify that the two groups were comparable for age, diabetes, and hypertension as well as for preserved residual function, hemoglobin and creatinine clearance at the onset of dialysis. We can postulate that twice weekly dialysis regimen can be a suitable schedule at the initiation of hemodialysis. In fact, almost half a century ago, the thrice-weekly HD schedule was scientifically established as a means to provide an adequate dialysis dose and to treat the great number of ESRD patients using limited resources [3]. The twice-weekly HD has been used for selected patients and it is still a current and common practice in South-east Asia [6]. Yet, in USA, K. Kalantar-Zadeh advocates an incremental regimen with 2 sessions per week when starting hemodialysis [12-15]. This permits the preservation of residual kidney function and so it has better results. By contrast, some authors disagree with this conception. They have compared clinical results between twice-weekly and thrice-weekly hemodialysis in patients with RKF. They found that the twice dialysis patients showed an independent association with the great risk of mortality compared to those with RKF undergoing thrice-weekly HD because of the low protein catabolic rate. Overall, they argue that the determination about twice-weekly HD should consider not only RKF but also other risk factors such as normalized protein catabolic rate [16, 17].

On the other hand, some other authors have criticized the ideas of K. Kalantar-Zadeh claiming that the preservation of residual renal function depends on low ultrafiltration rate in dialysis and so on the existence of low interdialytic weight gain, something difficult to achieve with twice weekly dialysis. Moreover, if the IDEAL study has confirmed starting dialysis on clinical symptoms, few patients will fulfill the criteria of including twice weekly regimen [18]. Paradoxically, few studies indicate that once-weekly HD regimen could be a viable starting option as well [6]. Kamyar Kalantar-Zadeh even suggests once-weekly hemodialysis combined with low-protein diet when starting hemodialysis. Such a gradual transition with less frequent hemodialysis sessions at the beginning and with progressive increase in hemodialysis frequency over months would also enhance a less stressful adaptation of the patient to dialysis therapy along with the favorable clinical and economic implications [19]. This dialysis modality is important and should be taken under consideration in developing countries where the number of patients who need dialysis is more than the number of available places. Certainly, we belong to a developing country and it is true that dialysis is widely available and it is well done but public units are overcrowded and we sometimes find ourselves compelled to treat some patients who don´t have social coverage once or twice a week. Indeed, our results are similar to others in many countries and they might encourage some African countries in particular, where HD is still in its starting point, to introduce twice weekly dialysis sessions if access to dialysis is limited.

## Conclusion

It is evident that a low dialysis dose in case of lack of means is hazardous. In the other hand, intensive hemodialysis with thrice weekly regimen might contribute to faster RKF loss and could lead to high mortality. So twice weekly dialysis can be a suitable option at the onset of dialysis. The key reason to favor twice than thrice weekly regimen is the preservation of residual kidney function which is the major predictor of survival. In summary, we assume that starting hemodialysis twice per week might be safe and could promote an economic management of incident hemodialysis patients mainly with the outbreak of the chronic renal failure. The challenge is improving survival especially in the critical period that succeeds the start of hemodialysis. Last but not least, we recognize also the importance of clinical trials to examine the safety and effectiveness of this hemodialysis regimen.

### What is known about this topic

- Mortality is high at the start of renal replacement therapy in low, middle and high-income countries;
- Incremental hemodialysis preserve native kidney function and may lead to better survival.

### What this study adds

- The twice or the threefold weekly hemodialysis treatment have similar outcomes on the patient’s survival (for 6 months);
- Twice-weekly hemodialysis can be safe at the beginning of kidney replacement therapy.

## Competing interests

The authors declare no competing interests.
